# Improving integrated depression and non-communicable disease care in Malawi through engaged leadership and supportive implementation climate

**DOI:** 10.1186/s12913-023-10344-7

**Published:** 2023-12-14

**Authors:** Griffin M. Sansbury, Brian W. Pence, Chifundo Zimba, Juan Yanguela, Kelsey Landrum, Maureen Matewere, MacDonald Mbota, Jullita K. Malava, Harriet Tikhiwa, Abigail M. Morrison, Christopher F. Akiba, Bradley N. Gaynes, Michael Udedi, Mina C. Hosseinipour, Melissa A. Stockton

**Affiliations:** 1 Tidziwe Centre, University of North Carolina Project-Malawi, Private Bag A-104, Lilongwe, Malawi; 2 Department of Epidemiology, Gillings School of Global Public Health, University of North Carolina at Chapel Hill, 135 Dauer Drive, 2101 McGavran-Greenberg Hall, CB #7435, Chapel Hill, NC 27599-7440 USA; 3 Department of Health Policy and Management, Gillings School of Global Public Health, University of North Carolina at Chapel Hill, 135 Dauer Drive, 1101 McGavran-Greenberg Hall, CB #7411, Chapel Hill, NC 27599-7411 USA; 4 Malawi Epidemiology and Intervention Research Unit (MEIRU), P.O. Box 46, Chilumba, Karonga District, Malawi; 5 RTI International, 3040 East Cornwallis Road, P.O. Box 12194, Research Triangle Park, NC 27709-2194 USA; 6 Division of Global Mental Health, Department of Psychiatry, University of North Carolina at Chapel Hill School of Medicine, 101 Manning Dr #1, Chapel Hill, NC 27514 USA; 7 Malawi Ministry of Health and Population, Non-Communicable Diseases and Mental Health Clinical Services, P.O. Box 30377, Lilongwe, 3, Malawi; 8 Department of Medicine, University of North Carolina at Chapel Hill School of Medicine, 101 Manning Dr #1, Chapel Hill, NC 27514 USA

**Keywords:** Leadership, Implementation climate, Mental health, Task-shifting, Sub-Saharan Africa, Qualitative research

## Abstract

**Background:**

Low- and middle-income countries often lack access to mental health services, leading to calls for integration within other primary care systems. In sub-Saharan Africa, integration of depression treatment in non-communicable disease (NCD) settings is feasible, acceptable, and effective. However, leadership and implementation climate challenges often hinder effective integration and quality of services. The aim of this study was to identify discrete leadership strategies that facilitate overcoming barriers to the integration of depression care in NCD clinics in Malawi and to understand how clinic leadership shapes the implementation climate.

**Methods:**

We conducted 39 in-depth interviews with the District Medical Officer, the NCD coordinator, one NCD provider, and the research assistant from each of the ten Malawian NCD clinics (note one District Medical Officer served two clinics). Based on semi-structured interview guides, participants were asked their perspectives on the impact of leadership and implementation climate on overcoming barriers to integrating depression care into existing NCD services. Thematic analysis used both inductive and deductive approaches to identify emerging themes and compare among participant type.

**Results:**

The results revealed how engaged leadership can fuel a positive implementation climate where clinics had heightened capacity to overcome implementation barriers. Effective leaders were approachable and engaged in daily operations of the clinic and problem-solving. They held direct involvement with and mentorship during the intervention, providing assistance in patient screening and consultation with treatment plans. Different levels of leadership utilized their respective standings and power dynamics to influence provider attitudes and perceptions surrounding the intervention. Leaders acted by informing providers about the intervention source and educating them on the importance of mental healthcare, as it was often undervalued. Lastly, they prioritized teamwork and collective ownership for the intervention, increasing provider responsibility.

**Conclusion:**

Training that prioritizes leadership visibility and open communication will facilitate ongoing Malawi Ministry of Health efforts to scale up evidence-based depression treatment within NCD clinics. This proves useful where extensive and external monitoring may be limited. Ultimately, these results can inform successful strategies to close implementation gaps to achieve integration of mental health services in low-resource settings through improved leadership and implementation climate.

**Trial registration:**

These findings are reported from ClinicalTrials.gov, NCT03711786. Registered on 18/10/2018. https://clinicaltrials.gov/ct2/show/NCT03711786.

**Supplementary Information:**

The online version contains supplementary material available at 10.1186/s12913-023-10344-7.

## Background

Depression drives morbidity and mortality globally, particularly in low- and middle-income countries (LMICs) [1–3]. This burden will increase as sub-Saharan Africa (SSA) undergoes an epidemiologic transition from transmissible to non-communicable diseases (NCDs) [4]. Depression is a common comorbidity in patients with hypertension, diabetes, and other NCDs, resulting in worse outcomes [5]. In Malawi, the prevalence of depression among diabetic adults is approximately 18% and mental health disorders are among the leading causes of NCD burden [6, 7]. As such, there are calls to integrate mental health treatment into NCD care systems in LMICs, such as Malawi, to provide equitable, accessible, affordable, and timely mental and NCD care [8, 9].

Despite calls for integration, mental health service scale-up in LMICs is limited by staff, resources, and other health system capacity constraints [10]. In SSA, the median number of mental health professionals per capita is 50 times lower than in high-income countries [11]. Consequently, more than 75% of patients with depression receive no treatment [7]. To bridge this gap, task-shifting approaches are a potential solution to expand mental healthcare access [12]. Task-shifting for mental health, an evidence-based implementation strategy, involves equipping primary care providers and community workers with the skills to deliver care for common mental disorders [13–16]. Trials conducted in SSA suggest that evidence-based practices (EBPs) for treating depression, including psychological counselling and algorithm-based antidepressant management, can successfully be task-shifted to non-specialists [17–19]. However, scale-up is hindered by barriers to wide-spread implementation and fidelity, including low self-perceived clinician competence, job-related stress, and lack of compensation for additional tasks related to mental health care [20].

Leadership and implementation climate are two vital factors for EBP deployment [21–25]. Leadership engagement is defined as leaders’ commitment, participation, and accountability with an intervention [26–30], while implementation climate relates to the degree to which the intervention is supported, rewarded, and expected within an institution or organization [31]. Emerging evidence suggests that leadership can act as both a facilitator of and a barrier to mental health EBP implementation [32, 33]. However, the evidence base is limited, as available studies suffer from poor methodological quality and fail to evaluate the conditions under which leadership affects implementation [33]. More rigorous, theory-informed research is essential to elucidate the impact of leadership (and its relationship with implementation climate) on implementation processes and outcomes in LMICs.

Using post-implementation data from the Sub-Saharan Africa Regional Partnership (SHARP) parent trial (NCT03711786), we sought to identify discrete leadership strategies that facilitate overcoming barriers to the integration of depression care in NCD clinics in Malawi and understand the role of clinic leadership in shaping the corresponding implementation climate.

## Methods

### Parent trial

SHARP was a clinic-randomized control trial that integrated evidence-based depression screening and treatment into NCD care in Malawi while comparing the effectiveness of two implementation strategies to improve implementation and patient outcomes. In partnership with the Malawi Ministry of Health (MOH), the trial was conducted in 10 geographically representative NCD clinics in Malawi from May 2019 to June 2022 [34].

### Depression screening and treatment program

The EBP task-shifted depression screening and treatment to NCD clinicians and nurses [34]. These non-specialists were trained to screen adult diabetes and/or hypertension patients for depression using the Patient-Health Questionnaire-9 (PHQ-9), a screening tool previously validated in this setting [35]. Patients with mild depression (PHQ-9 score 5–9) were to be referred to the Friendship Bench, a culturally adapted, problem-solving therapy delivered by trained peer experts through one-on-one counselling [17]. Those with moderate to severe depression (PHQ-9 score ≥ 10) were prescribed antidepressants (fluoxetine or amitriptyline) according to measurement-based care (MBC), an algorithm-guided antidepressant management protocol in line with current treatment practices [19, 36]. In-depth descriptions of the depression program have been published [34, 37–39].

### Study design

This endpoint qualitative analysis focused on the manifestation and impact of leadership and implementation climate on overcoming clinic barriers, a theme identified, but not extensively explored, in a midpoint analysis [39]. These data expand our understanding of these two themes in the Malawian mental health landscape by including all clinic sites in analysis and with the benefits of hindsight at the study’s completion.

### Study sites

The SHARP trial was conducted at 10 NCD clinics located in MOH secondary-level district and community hospitals. The participating NCD clinics spanned the three regions of Malawi (northern [*n* = 2], central [*n* = 4], and southern [*n* = 2]).

### Participants

In-depth interviews (IDIs) were conducted with 39 participants that included the District Medical Officer (DMO), the NCD coordinator, one NCD provider, and the SHARP research assistant (RA) from each of the 10 study sites(two district hospitals shared one DMO). Purposive sampling was utilized to identify participants who were approached via face-to-face interactions or telephone interactions. DMOs were targeted due to their leadership status and decision-making power to solve implementation issues at the NCD clinic-level. NCD coordinators managed day-to-day integration activities and had extensive knowledge of implementation processes. NCD providers adopted depression screening and treatment into the routine practice and interacted closely with DMOs and NCD coordinators; notably they were identified by NCD coordinators. SHARP RAs were non-clinical study staff working in the sites, but not part of the MOH clinic structure and therefore less likely to be biased by positive responses. In comparison, DMOs, NCD coordinators, and NCD providers were all formally employed by the MOH.

### Data collection

Data from qualitative, semi-structured IDIs were collected during the SHARP trial from November 2021 to January 2022 to contextualize and assess the role of leadership and implementation climate in overcoming barriers to integration of depression care into NCD clinics in Malawi. Interviews also explored the impact of individual leaders’ engagement on integration success, the power of program champions to motivate providers, and the manifestation of teamwork. These findings facilitate the discovery and analysis of contextual factors surrounding program implementation from the implementers’ perspective [40]. The complete interview guides can be found in the Supplementary Material.

SHARP RAs interviewed the other participant groups at a site different from their placement. An interviewer not involved with the parent trial interviewed the SHARP RAs. Most (*n* = 34) IDIs were conducted over the phone due to COVID-19 in a location of the participant’s choosing. Some (*n* = 5) IDIs were conducted in private locations in the respective district hospitals. The 1-h interviews were conducted in English, with some participants adding occasional words or sentences in Chichewa, a local dialect in Malawi. Interviews were recorded, transcribed verbatim, and translated as necessary using a one-step approach.

The study team used deductive and inductive analyses to develop a codebook relevant to the main objectives of the endpoint qualitative study and to capture emerging themes [41, 42]. Parent codes were developed based on interview guides and primary study aims while child codes represented emergent themes and study sub-aims. Additionally, study team members reviewed the transcripts to provide feedback to the interviewers and ensure appropriate application and iterative adaption of the guides.

### Data analysis

Seven team members coded the transcripts using NVivo 12. To establish inter-coder reliability and iteratively update the codebook, the team met frequently to review application of the pre-determined codes and add new codes based on emergent themes. Initially, the entire team coded the same two transcripts before conducting a line-by-line analysis, adjudicating coding discrepancies, and adapting the codebook where needed. Thereafter, two team members coded the same transcripts with the goal of 80% agreement through inter-coder reliability assessment and adjudication of any coding discrepancies.

Researchers used matrices to summarize transcripts and aid in thematic analysis. These matrices allowed the team to thematically organize the data, deepen understanding of key themes, and compare themes amongst participants, participant groups, and study sites [43]. Researchers created memos to summarize key themes such as participant perceptions of leadership, their roles in integration, and their power in increasing intervention fidelity [44, 45]. The Consolidated Framework for Implementation Research guided further processing of data, specifically focusing on leadership with respect to the following sub-constructs: implementation climate, intervention source, relative priority, goals and feedback, learning climate, leadership engagement, access to knowledge and information, knowledge and beliefs about the intervention, self-efficacy, individual identification with the organization, and opinion leaders [46].

## Results

### Participants

The leadership organization of NCD clinics in Malawi is described in Table 1 to delineate hierarchy among the participants, along with participants’ intended role in the project.

**Table 1:** Levels of NCD leadership in Malawi and job descriptions

Job Title	Jurisdiction	Qualifications	Job description	Intended role in depression integration	Number of participants
MOH Official	National level	Masters or PhDs	Oversee and implement Ministry of Health clinical initiatives	Ensure smooth functioning of the depression program. Transfer service providers between clinics, supply standard operating procedures and protocols, enhance ownership for integration	^a^
DMO	District hospital level	Physicians	Responsible for all clinical care at the district hospital and directly supervise all department coordinators	Oversee integration activities and respond to clinic challenges through interaction with monthly reports and collaborating with NCD coordinators, most notably by staffing NCD clinics and ensuring antidepressant medication supply	9
NCD Coordinator	Department level	Physicians, physician assistants/clinical officers, or nurses Appointed by the MOH	Responsible for NCD clinic care at the district hospital and at lower facilities within the district. Directly oversees NCD providers at district level	Training and supervising all facility healthcare workers in the depression program, scheduling and record keeping, communicating implementation challenges with facility leadership (DMO), and submitting monthly reports to the DMO/MOH	10
NCD Provider	Clinic level	Physician assistants/clinical officers or nurses	Provide clinical care to NCD patients	Responsible for screening of depression, referral to Friendship Bench therapy, and prescription of antidepressants according to MBC	10
SHARP RA	Research activities at clinic level	University degrees or diplomas in public health	_ _ _	Consent and survey participants and abstract clinic data. Note, they do not play a direct role in the facilitation of the depression program	10

^a^MOH officials were not participants, but played important roles in integration and were referenced as ‘leaders’ in the interviews

The results section is organized by a presentation of themes related to specific leadership strategies, and lack thereof, that both promoted a positive implementation climate and facilitated overcoming barriers to depression integration. The following key themes are explored in detail: engagement and approachability; direct involvement with and mentorship during the intervention; clear communication; and fostering teamwork.

### Engagement and approachability

Engaged leaders were aware of integration barriers, involved in strategizing solutions, and active in implementing clinic changes. Initially, integration barriers, such as being overburdened by a heavy workload, medication shortages, and negative attitudes by NCD providers, contributed to sub-optimal depression screening for NCD patients at most district hospitals. However, many study participants reported that DMOs effectively problem-solved through heightened awareness of clinic challenges and directed follow-up based on metrics from quarterly reports sent by NCD coordinators. This bred a positive working environment and led to tangible changes to existing integration barriers, as illustrated by one NCD provider’s account of clinic congestion:

“At first we used to do NCD services in the corridors and they [the DMO] directed that we should move from the corridors because of congestion…So them as the DMO told us to move to the skin clinic and you know the skin clinic occurs on Monday and Fridays and we do our NCD on Thursday. So by moving the NCD to the skin department it means that they are aware of the challenges that we face in providing depression care integration.”

– NCD Provider

Through awareness of limited space, the aforementioned DMO effectively relocated integration activities to a private location. Through implementing this change, reported clinic functioning and integration outcomes improved at this NCD clinic. This DMO sustained involvement with clinic operations and overcoming barriers, even congratulating the study team in front of the MOH and program evaluation team. By directly acknowledging the clinic’s successes and providing positive reinforcement to the study team in front of the MOH, this DMO fostered a supportive learning climate where providers felt valued for their contributions.

However, not all DMOs were perceived as engaged and approachable. Two facilities indicated a negative working relationship with their DMO due to a lack of follow-up with reports and an authoritarian leadership style. One NCD coordinator described an incident where they proposed increasing the number of NCD clinic days to distribute a high workload burden. They describe the DMO’s response below:

“Unfortunately, it was like a directive that ‘myself as the DMO I am not even asking you and I am not suggesting but I am saying one clinic a day’…we thought the decision would really worsen the situation because if we were having congestion on two days, it means the number of people in one day will double…it means we would end up leaving a lot of people not being screened. So sentiments like these meant that the line managers [DMOs] don’t have much interest in the program.”

– NCD Coordinator

Through what participants reported as an inflexible perspective and belittling attitude, this DMO failed to (1) acknowledge the NCD coordinator’s request; (2) validate their feelings of being overworked; and (3) use their decision-making power to solve a structural problem. NCD coordinators and providers perceived this precedent of being unwilling to collaborate to signify that district and national leaders did not place much relative priority on depression treatment integration, leading clinicians to feel less motivated to take an active role in depression screening.

Although perceptions of DMOs were district hospital specific, at district hospitals that reported a positive working relationship with their DMO, almost all also reported a subsequent improvement in integration outcomes and cultivation of a supportive implementation climate. DMOs who established good rapport were more likely to be approached and welcomed by clinic staff. Clinicians were empowered through their healthy relationship with the DMO to openly call them in times of need:

“Mostly when we call the DMO they pick up our phones and if they are busy and has just seen your missed call they might call you afterwards to know why you were calling. That’s why I am saying there is a good rapport or interaction between [us] coordinators and the DMO.”

– NCD Coordinator

Through maintaining open lines of communication, this DMO instilled within the NCD coordinator a sentiment of being an integral member of the implementation team, which sustained their motivation to screen and provide integration services. Other NCD coordinators and providers described having a “real partner” in integration with the DMO and MOH following sustained engagement with these influential leaders, breeding a positive implementation climate.

### Direct involvement with and mentorship during the intervention

Providers generally agreed that direct leadership supervision and support helped maintain fidelity to the depression screening protocol because providers knew their work was being scrutinized. This support was accomplished through top-down leadership, whereby higher-ranking MOH officials and DMOs supervised middle-tiered NCD coordinators, and all leaders oversaw NCD providers.

Some participants reported that the MOH was able to leverage its position of power by physically visiting district hospitals and establishing the importance of the intervention in the clinic and larger healthcare system. NCD coordinators were highly motivated by such visits from MOH officials:

“They [the MOH] support us with supportive supervision, they come and see what we are doing, and it is really a Ministry of Health initiative. Partners are there to guide and support us. It is a learning point as a study so that others can emulate and can learn some stuff so that they can scale up to other…but it is solely a Ministry of Health…They are like our consultants.”

– NCD Coordinator

NCD coordinators and providers alike sustained personal responsibility for integration as they interpreted the MOH officials’ visits to reaffirm their involvement in the interaction. By viewing the program as a “*Ministry of Health initiative*,” clinicians understood that their work would inform future scale-up efforts. Additionally, as NCD coordinators and providers learned from MOH expertise and consulted with them on difficult cases, their innate connection and value tied to the intervention increased, further enhancing clinic ownership.

DMOs were similarly perceived to motivate clinicians through visibility due to respect associated with their position. One NCD coordinator depicted that,

“Because if the DMO comes it gives us encouragement to do better because we know that if the big man is here they need to see that our operation is just so good…we know that we need to do things in a right manner.”

– NCD Coordinator

By describing the DMO as a *“big man,”* this NCD coordinator acknowledges a power dynamic whereby higher-ranking leaders were able to command respect and positively motivate integration through their status. These routine visits by MOH officials and DMOs were able to keep team members engaged, as their presence in clinic demanded the need for consistent depression screening and care *“in a right manner.”*

However, this unique ability to invoke motivation through visibility alone could not be recreated by NCD coordinators, leaders who worked in the clinic every day and with whom providers had much familiarity.

“I do not think that the coordinator has some special powers that they may be able to influence others to screen each and every patient who comes to the NCD clinic…because the position they have is not an employed position, it is an appointed position, so they are of the same rank as the other clinicians. So, they do not have that much power to maybe do some supervisions that in some way they might be able to inspire maybe some fear in their fellow clinicians. But, if maybe they were of a higher rank… I think then they could have been able to inspire others to do their work as they are supposed to do.”

– RA

The RA describes how the power dynamic between NCD coordinators and NCD providers is not as stark compared to the MOH and DMOs with NCD providers. NCD coordinators, in some instances, were unable to leverage their position to influence screening due to similar levels of training and associated status with NCD providers. Instead, leaders with significantly more training and authority possessed heightened capacity to motivate the cadres due to respect associated with higher standing, and, occasionally, through invoking fear.

All leaders, regardless of position, acted as opinion leaders when leading by example, which positively affected integration. Whenever DMOs or NCD coordinators physically took part in depression screening and treatment, they both mentored NCD providers and modelled ideal behaviour. As a result, NCD providers imitated leaders’ attitudes towards integration and mirrored their actions in actively screening for depression. Imitation was a dominant theme, as almost all NCD providers noted increased motivation for depression screening when NCD coordinators were modelling ideal behaviour. This sense of communal commitment to the intervention was described as follows:

“It’s quite helping enough because when we see our boss [NCD coordinator] in the forefront it’s quite difficult for us to do the opposite…since they act as a leader, they lead us on what to do and we just follow suit…they are directly affecting depression clinic in a positive way. Yeah I would say their presence is always a plus for us and the way they handle depression cases that’s also what motivates us to try to do our best as far as depression screening and treatment.”

– NCD Provider

Although NCD coordinators could not invoke fear, they influenced screening through camaraderie and working *“in the forefront”* alongside providers. Thus, NCD coordinators were influential mentors from which providers could learn and imitate (*“follow suit”*) their attitudes and behaviours. NCD coordinators’ commitment to actively integrating depression care proved to be the most impactful in changing NCD provider attitudes and habits. When asked who has the power to address barriers, specifically negative attitudes by providers, a majority of participants identified their NCD coordinator due to their coordinator’s availability and involvement. Engaged mentorship also fostered a positive implementation climate that championed individual commitment to the intervention, praising NCD providers who gained a deeper understanding of MBC and took ownership of their work.

### Clear communication

Clear communication was a key motivating factor for depression care integration. Effective leaders were able to clarify specific misconceptions or misunderstandings to increase NCD provider knowledge and commitment to MBC. At facilities where misconceptions about the source of the intervention and financial compensations were held, clinic staff noticed NCD providers’ reluctance to screen patients:

“For this thing to work [integration of depression and NCD care], we need to have a mindset change from the providers, managers, as well as the policy makers. So prioritization becomes a challenge because they [NCD providers] do prioritize some of the other programs over this program…people would have some different attitudes, people would have different perceptions…they would think that maybe the [NCD] coordinator is benefitting something and people would think that the program is for the [NCD] coordinator.”

– NCD Coordinator

At this district hospital, NCD providers became less interested and disinclined to follow MBC because of a perception that NCD coordinators were benefitting financially from the depression integration intervention when they were not. This further demotivated clinical staff, who even started to “*prioritize some of the other programs over this program*.”

At a different district hospital facing similar pushback from NCD providers, the NCD coordinator engaged MOH officials and DMOs for clarity around incentives and sustainability, stating:

“The Head of Department [MOH/DMO] has been emphasizing on this as a service from the Ministry of Health…any clinician, any nurse who does not respond is answerable not to the project but the Ministry of Health, directly to the Head of Department. So to us this is not a project, it is a service. [The external research entity] is just supporting what the government is doing.”

– NCD Coordinator

Through engaging policy makers, like MOH officials, and establishing a clear chain of communication from the intervention source to implementing NCD providers, misconceptions about external research project support were curbed. In establishing MOH responsibility for noncompliance, leaders enhanced clinic ownership of the program. Even at district hospitals facing less provider resistance, clarifying the depression program as a sustainable, government-derived initiative increased NCD provider’s understanding and commitment to MBC. NCD providers themselves recognized the importance of this chain of communication stating, “*also any information coming from top going down should come in time and be sufficiently communicated to* [us].”

Another reason NCD providers were not fully committed to MBC at select district hospitals stemmed from a lack of value placed on mental health and the intervention itself. Some NCD providers did not understand the psychosocial aspects of NCD care nor recognize that NCD patients even faced mental health challenges. As a result, providers were less likely to follow MBC. To demonstrate support for the intervention, leaders, like NCD coordinators, educated providers on the value of mental healthcare,

“We usually tell them [NCD providers] that if you don’t help these patients, if we don’t find cases, we may end up losing due to depression itself or suicide ideation. So, we advise them to have a passion to the patients to make sure that they are assisted…so by doing that the screening is done most of the times…we talk to them in morning reports that is for the whole hospital, the whole team, about the consequences, the negative impact of depression.”

– NCD Coordinator

This NCD coordinator explained why the MOH supports integration through digestible knowledge about the impacts of mental disorders on chronic care patients, most notably by describing suicidal ideation. By contextualizing the work through a patient perspective, leaders and providers became intrinsically motivated for mental health improvements and passionate about integration activities through shared compassion and empathy for patients. This is seen through NCD providers’ recommendations to expand depression services into other clinical sectors.

“I would wish in the first place… like every patient who came to the hospital… whether they had a wound, whether they just complained of headache or had diarrhea but is seeking medical intervention should be screened for depression. I think with that we have seen a lot of patients who have so far committed suicide. In a sense right now we are missing a lot of depressed patients because we are only screening patients who have come to the hospital because of diabetes or hypertension, but had it been we were screening every patient we would have found a lot of them. Had it been it was preached a lot to the community we would have found a lot of depressed patients.”

– NCD Provider

This NCD provider exemplified the impact education on mental health had on individual attitudes towards depression integration. By recognizing (1) the prevalence of and (2) the under diagnosis of depression in their community, this NCD provider demonstrated heightened awareness of the public health impact of their work. Their desire to increase access to depression services at their district hospital also suggested personal satisfaction and efficacy with the depression program.

### Fostering teamwork

Sites with more involved DMOs benefitted from stronger teamwork and clinic functioning. However, regardless of clinic performance or DMO engagement, most participants identified teamwork as an important component of integration. Participants described teamwork as the cooperation of NCD providers within a clinic and engagement among national-, district-, and hospital-level leaders. Such leaders fostered team spirit through their support and visibility in the district hospital and by improving team morale, strength, and cooperation.

“In level of support I’d say yes since the DMO is there to support us. I’d say that it's not that difficult for us to involve other clinicians because there is someone there at the top who is really supporting us and who is understanding what is happening to them. So them being there to support us at the clinic, this has given us morale to push on… despite the challenges that we have. It gives us that strength to work as a team.”

– NCD Coordinator

Working in teams signified communal strength and morale to overcome integration barriers. Team success was largely dependent on collective responsibility for the intervention, especially at sites that had greater collaboration and task-sharing.

“It’s a collective responsibility; one, the individuals themselves to understand what is required and also to make sure to accept the adjustments in the clinic; and two the constant mentoring at the clinic has also helped us much.”

– DMO

Through mentorship and hands-on engagement, leaders infused collective responsibility and team spirit, whereby providers were motivated to work alongside leaders. At sites where the DMO and NCD coordinator contributed to integrating depression care, this lessened the workload for any individual provider, leading to positive attitudes and higher intervention fidelity.

Working in teams also facilitated effective decision-making and incorporation of individuals’ suggestions to overcome barriers and improve clinic functioning.

“We sit down as a team because you cannot work alone, we sat down and discussed as a team… Yea so we sit down as a team when we have a problem and say ‘this is a problem so as a team what do you think we can do to solve this problem?’ So, our colleagues proposed that maybe we can open another day. So, we agreed that ‘let’s just open another day,’ so that’s what we do. We had to sit down as a team not just one man’s show because nowadays there is freedom and we need to discuss and have a solution.”

– NCD Coordinator

With this open perspective towards the importance of teamwork, this NCD coordinator constructed a respectful learning climate that fostered collective team assistance and input. As an engaged and approachable team leader, the NCD coordinator validated provider concerns, collectively strategized solutions, and adapted the implementation strategy to more effectively screen for depression.

## Discussion

We investigated key implementers’ perspectives on leadership strategies to enhance the implementation climate surrounding and fidelity to integrating a depression treatment program into existing NCD care in Malawi. Some sites implemented these leadership strategies better than others and thus reported improved implementation outcomes. Alignment among clinic leaders, through engagement, approachability, and awareness of clinic challenges, fostered a learning environment conducive to implementation success. Participants noted the hierarchies inherent in the Malawian healthcare system and their corresponding effect on the implementation climate. Most participants emphasized mentorship and hands-on learning as key leadership strategies that created a productive implementation climate. NCD providers could observe, engage with, and collaborate alongside leaders, most notably NCD coordinators, when they were physically present and modelling ideal screening and clinical behaviour.

Alignment among facility and clinic leaders with implementing NCD providers was crucial in problem solving clinic challenges and ensuring changes were implemented and effectively improved fidelity. These findings are consistent with various implementation frameworks and interventions that stress aligning priorities among different levels of leadership [46–51]. Furthermore, they highlight the importance of “leader inclusiveness,” defined as “*words and deeds exhibited by leaders that invite and appreciate others’ contributions*” [52]. DMOs and NCD coordinators who explicitly invited input from NCD providers and provided positive reinforcement were reported to be more engaged with the intervention and more adept at fostering active participation and commitment from providers. Literature suggests such leaders strengthen communication systems, which promote effective EBP implementation [53]. Some leadership frameworks and interventions have been utilized for mental health task-shifting services [47] and adapted for different cultural and contextual settings [54]. Thus, future task-shifting programs in SSA should consider incorporating such frameworks to improve alignment among leadership to overcome integration barriers common to this implementation landscape.

Understanding leadership context and culture is necessary for adapting and implementing effective leadership strategies to improve implementation climates and outcomes [55, 56]. Unlike other implementation frameworks that describe broad domains of leadership behaviours, we identified specific behaviours that are pragmatic for different levels of leadership, so leaders can tailor their unique training, experience, and influence to support EBPs [50, 57–59]. In Malawi, a hierarchy exists in the healthcare system from the NCD providers’ perspective. High-ranking MOH officials and DMOs could support integration solely through visibility during “*fear*”-inspiring quality improvement checks that championed provider accountability. In contrast, clinical leadership, in the form of NCD coordinators, had to display long-term commitment to and physically be involved in depression management procedures to influence provider attitudes. Underscoring these differing attitudes towards leadership was respect associated with status and increased level of training. Thus, an unbalanced power dynamic between different leaders meant MOH officials and DMOs had the authority to be less involved than NCD coordinators while still maintaining the ability to reinforce screening behaviours and instil clinic ownership. As there is limited literature on leadership behaviours in LMICs and in SSA, these results suggest understanding the healthcare system as a whole – and the unique levels of leadership within that system – is necessary to tailor implementation strategies to engage and leverage the different levels of leadership.

Some DMOs and NCD coordinators overcame barriers to integration by educating providers on the high prevalence of comorbid depression and NCDs, the adverse impact of depression, and the value of the intervention. NCD providers noted the positive impact when NCD coordinators used evidence (e.g., statistics) to contextualize mental health education. This education improved the implementation climate as providers understood the public health importance of the intervention and were intrinsically motivated to screen and treat patients. Additionally, this heightened awareness led NCD providers to desire increased access to depression screening and treatment for other departments within the district hospital. These results speak to the importance of mental health literacy for all providers, a key determinant of both the provision and accessibility of mental health services [60, 61]. Integration of mental health services in primary care settings in SSA has been hampered by negative provider attitudes and low prioritization of mental healthcare [32]. In Malawi, a cross-sectional survey demonstrated that a majority of both providers and patients had limited mental health understanding, attributing causes of mental disorders to substance use or spiritual “punishment” [62]. As low mental health literacy exacerbates treatment gaps in the region, transformational leaders’ ability to improve provider attitudes towards mental healthcare could serve as a valuable intervention [63]. Thus, engaging leadership to improve mental health literacy, address stigma, and underscore the public health importance of providing mental healthcare could improve the implementation climate for task-shifted mental health programming.

Direct mentorship by leaders was key to educating NCD providers on best practices for MBC. NCD providers worked alongside and shadowed more experienced DMOs and NCD coordinators to gain on-the-job refresher training and consultation on complex cases. Most participants emphasized that this mentorship is vital for enhancing confidence in MBC and sustained intervention fidelity. Conversely, without this hands-on support and supervision, providers may not have screened patients with the same rigor due to a lack of self-efficacy. In SSA, insufficient resources for mental health treatment, often compounded by low mental health literacy, undermines providers’ ability to consistently deliver mental health treatment and reinforce negative attitudes [63–65]. However, mentoring programs can effectively increase health worker competence and institutional performance for a variety of clinical specialties [66]. Furthermore, in SSA, mentorship may also be superior to refresher trainings as these trainings are limited in frequency, scope, and often didactic rather than interactive in nature [67]. Together, our results support enhanced leadership strategies that incorporate engaged, on-the-job mentorship, although cultural and healthcare system contextualization should dictate the mentorship approach [66].

### Limitations

Participants were all involved in some respect in the implementation and/or evaluation of this intervention and trial, and their shared reflections on the challenges and successes of the program may have been subject to social desirability bias. Though we cannot reduce the risk of social desirability bias entirely, including study RAs as participants (whose views may be less biased than other participants) is a critical measure to reducing this bias in our study. A small convenience sample of healthcare facility staff from the study sites participated in the interviews and their views might not be generalizable to all study site staff. However, the sample did include participants from all key levels of leadership, implementation, and evaluation and from all ten study sites, enabling researchers to elicit a wide variety of perspectives. Lastly, we analysed participant responses without regard to the implementation strategy their site received. We believe by examining implementation challenges and leadership strategies that potentially diffused to most district hospitals we more wholly captured the reality of this implementation landscape.

## Conclusions

Leadership-driven implementation strategies may improve task-shifting of mental health services. However, few studies in LMIC have examined or defined how to leverage leadership and maintain research capacity capable of addressing mental healthcare gaps [68]. Researches and implementers must understand how to adapt not only the EBP, but also the implementation strategy to fit the existing healthcare system and implementation climate [69]. This qualitative study contributes to the nascent body of literature in SSA on the healthcare structure and implementation climate surrounding mental health task-shifting interventions. We highlighted specific ways leadership can improve intervention fidelity, implementation outcomes, and the overarching implementation climate. Our results have implications for ongoing Malawi Ministry of Health efforts to scale up evidence-based depression treatment within existing NCD care. Insights from this study suggest tangible ways leaders can foster positive attitudes and personal responsibility to enhance fidelity and implementation for task-shifting interventions in low-resource settings. Future research should explore integrating leadership implementation frameworks and interventions with task-shifting mental health services to improve implementation and long-term research capacity.

### Supplementary Information

**Additional file 1.** Interview guide.

## Data Availability

The qualitative dataset is available on reasonable request to Griffin Sansbury at griffin.sansbury@alumni.unc.edu.

## Abbreviations

LMIC: Low- and middle-income countries
SSA: Sub-Saharan Africa
NCDs: Non-communicable diseases
EBPs: Evidence-based practices
SHARP: Sub-Saharan Africa Regional Partnership for Mental Health Capacity Building
MOH: Malawi Ministry of Health
PHQ-9: Patient-Health Questionnaire-9
MBC: Measurement-based care
IDIs: In-depth interviews
DMO: District medical officer
RA: Research assistant

## References

[1] Lopez AD, Mathers CD, Ezzati M, Jamison DT, Murray CJ (2006). Global and regional burden of disease and risk factors, 2001: systematic analysis of population health data. Lancet.

[2] Kessler RC, Aguilar-Gaxiola S, Alonso J, Chatterji S, Lee S, Ormel J, Ustun TB, Wang PS (2009). The global burden of mental disorders: An update from the WHO World Mental Health (WMH) Surveys. Epidemiol Psichiatr Soc.

[3] Demyttenaere K, Bruffaerts R, Posada-Villa J, Gasquet I, Kovess V, Lepine JP (2004). Prevalence, severity, and unmet need for treatment of mental disorders in the World Health Organization World Mental Health Surveys. JAMA.

[4] Charlson FJ, Diminic S, Lund C, Degenhardt L, Whiteford HA (2014). Mental and substance use disorders in Sub-Saharan Africa: predictions of epidemiological changes and mental health workforce requirements for the next 40 years. PLoS ONE.

[5] Moussavi S, Chatterji S, Verdes E, Tandon A, Patel V, Ustun B (2007). Depression, chronic diseases, and decrements in health: results from the World Health Surveys. Lancet.

[6] Udedi M, Pence BW, Stewart RC, Muula AS (2020). Detection and prevalence of depression among adult type 2 diabetes mellitus patients attending non-communicable diseases clinics in Lilongwe, Malawi. Int J Ment Health Syst.

[7] Frankish H, Boyce N, Horton R (2018). Mental health for all: a global goal. Lancet.

[8] Prince M, Patel V, Saxena S, Maj M, Maselko J, Phillips MR (2007). No health without mental health. Lancet.

[9] Raviola G, Becker AE, Farmer P (2011). A global scope for global health–including mental health. Lancet.

[10] World Health Organization. Accelerating country action on mental health. 2018. https://www.who.int/publications/i/item/9789240049338.

[11] World Health Organization. Mental Health Atlas 2011. 2011. https://www.who.int/publications/i/item/9799241564359.

[12] Rathod S, Pinninti N, Irfan M, Gorczynski P, Rathod P, Gega L (2017). Mental Health Service Provision in Low- and Middle-Income Countries. Health Serv Insights.

[13] MacArthur G (2008). Challenges and priorities for global mental health research in low- and middle-income countries.

[14] Baine SO, Kasangaki A (2014). A scoping study on task shifting; the case of Uganda. BMC Health Serv Res.

[15] van Ginneken N, Tharyan P, Lewin S, Rao GN, Meera SM, Pian J (2013). Non-specialist health worker interventions for the care of mental, neurological and substance-abuse disorders in low- and middle-income countries. Cochrane Database Syst Rev.

[16] O’Neil A, Jacka FN, Quirk SE, Cocker F, Taylor CB, Oldenburg B (2015). A shared framework for the common mental disorders and Non-Communicable Disease: key considerations for disease prevention and control. BMC Psychiatry.

[17] Chibanda D, Weiss HA, Verhey R, Simms V, Munjoma R, Rusakaniko S (2016). Effect of a primary care–based psychological intervention on symptoms of common mental disorders in Zimbabwe: a randomized clinical trial. JAMA.

[18] Pence BW, Gaynes BN, Atashili J, O'Donnell JK, Kats D, Whetten K (2014). Feasibility, safety, acceptability, and preliminary efficacy of measurement-based care depression treatment for HIV patients in Bamenda. Cameroon AIDS Behav.

[19] Wagner GJ, Ngo V, Goutam P, Glick P, Musisi S, Akena D (2016). A Structured Protocol Model of Depression Care versus Clinical Acumen: A Cluster Randomized Trial of the Effects on Depression Screening, Diagnostic Evaluation, and Treatment Uptake in Ugandan HIV Clinics. PLoS ONE.

[20] Padmanathan P, De Silva MJ (2013). The acceptability and feasibility of task-sharing for mental healthcare in low and middle income countries: a systematic review. Soc Sci Med.

[21] Ojo T, Kabasele L, Boyd B, Enechukwu S, Ryan N, Gyamfi J (2021). The Role of Implementation Science in Advancing Resource Generation for Health Interventions in Low- and Middle-Income Countries. Health Serv Insights.

[22] Corrigan PW, Lickey SE, Campion J, Rashid F (2000). Mental health team leadership and consumers satisfaction and quality of life. Psychiatr Serv.

[23] Aarons GA, Glisson C, Green PD, Hoagwood K, Kelleher KJ, Landsverk JA (2012). The organizational social context of mental health services and clinician attitudes toward evidence-based practice: a United States national study. Implement Sci.

[24] Green AE, Albanese BJ, Cafri G, Aarons GA (2014). Leadership, organizational climate, and working alliance in a children's mental health service system. Community Ment Health J.

[25] Skar AS, Braathu N, Peters N, Bækkelund H, Endsjø M, Babaii A (2022). A stepped-wedge randomized trial investigating the effect of the Leadership and Organizational Change for Implementation (LOCI) intervention on implementation and transformational leadership, and implementation climate. BMC Health Serv Res.

[26] Aarons G, Moullin J, Ehrhart M (2018). "The role of organizational processes in dissemination and implementation research" in Dissemination and Implementation Research in Health: Translating Science to Practic.

[27] Harvey G, Fitzgerald L, Fielden S, McBride A, Waterman H, Bamford D (2011). The NIHR collaboration for leadership in applied health research and care (CLAHRC) for Greater Manchester: combining empirical, theoretical and experiential evidence to design and evaluate a large-scale implementation strategy. Implement Sci.

[28] Kyratsis Y, Ahmad R, Hatzaras K, Iwami M, Holmes A (2014). Health Services and Delivery Research. Making sense of evidence in management decisions: the role of research-based knowledge on innovation adoption and implementation in health care.

[29] Nielsen K (2013). Review article: How can we make organizational interventions work? Employees and line managers as actively crafting interventions. Human Relations.

[30] Nielsen K, Randall R (2012). The importance of employee participation and perceptions of changes in procedures in a teamworking intervention. Work Stress.

[31] Weiner BJ, Belden CM, Bergmire DM, Johnston M (2011). The meaning and measurement of implementation climate. Implement Sci.

[32] Wakida EK, Talib ZM, Akena D, Okello ES, Kinengyere A, Mindra A (2018). Barriers and facilitators to the integration of mental health services into primary health care: a systematic review. Syst Rev.

[33] Meza RD, Triplett NS, Woodard GS, Martin P, Khairuzzaman AN, Jamora G (2021). The relationship between first-level leadership and inner-context and implementation outcomes in behavioral health: a scoping review. Implement Sci.

[34] Gaynes BN, Akiba CF, Hosseinipour MC, Kulisewa K, Amberbir A, Udedi M (2021). The Sub-Saharan Africa Regional Partnership (SHARP) for mental health capacity-building scale-up trial: study design and protocol. Psychiatr Serv.

[35] Udedi M, Muula AS, Stewart RC, Pence BW (2019). The validity of the patient health Questionnaire-9 to screen for depression in patients with type-2 diabetes mellitus in non-communicable diseases clinics in Malawi. BMC Psychiatry.

[36] Pence BW, Gaynes BN, Adams JL, Thielman NM, Heine AD, Mugavero MJ (2015). The effect of antidepressant treatment on HIV and depression outcomes: the SLAM DUNC randomized trial. AIDS (London, England).

[37] Akiba CF, Zimba CC, Thom A, Matewere M, Go V, Pence B (2020). The role of patient-provider communication: a qualitative study of patient attitudes regarding co-occurring depression and chronic diseases in Malawi. BMC Psychiatry.

[38] Zimba CC, Akiba CF, Matewere M, Thom A, Udedi M, Masiye JK (2021). Facilitators, barriers and potential solutions to the integration of depression and non-communicable diseases (NCDs) care in Malawi: a qualitative study with service providers. Int J Ment Heal Syst.

[39] Akiba CF, Go VF, Powell BJ, Muessig K, Golin C, Dussault JM (2023). Champion and audit and feedback strategy fidelity and their relationship to depression intervention fidelity: A mixed method study. SSM - Mental Health.

[40] Jack SM (2006). Utility of qualitative research findings in evidence-based public health practice. Public Health Nurs.

[41] Guest G, MacQueen K, Namey E. Applied Thematic Analysis. Thousand Oaks, California2012. Available from: https://methods.sagepub.com/book/applied-thematic-analysis.

[42] Elo S, Kyngäs H (2008). The qualitative content analysis process. J Adv Nurs.

[43] Hsieh HF, Shannon SE (2005). Three approaches to qualitative content analysis. Qual Health Res.

[44] Montgomery P, Bailey PH (2007). Field notes and theoretical memos in grounded theory. West J Nurs Res.

[45] Birks M, Chapman Y, Francis K (2008). Memoing in qualitative research: Probing data and processes. J Res Nurs.

[46] Damschroder LJ, Aron DC, Keith RE, Kirsh SR, Alexander JA, Lowery JC (2009). Fostering implementation of health services research findings into practice: a consolidated framework for advancing implementation science. Implement Sci.

[47] Aarons GA, Ehrhart MG, Farahnak LR, Hurlburt MS (2015). Leadership and organizational change for implementation (LOCI): a randomized mixed method pilot study of a leadership and organization development intervention for evidence-based practice implementation. Implement Sci.

[48] Aarons GA, Ehrhart MG, Moullin JC, Torres EM, Green AE (2017). Testing the leadership and organizational change for implementation (LOCI) intervention in substance abuse treatment: a cluster randomized trial study protocol. Implementation Sci.

[49] Birken SA, Lee SYD, Weiner BJ (2012). Uncovering middle managers' role in healthcare innovation implementation. Implementation Sci.

[50] Stetler CB, Ritchie JA, Rycroft-Malone J, Charns MP (2014). Leadership for evidence-based practice: strategic and functional behaviors for institutionalizing EBP. Worldviews Evid Based Nurs.

[51] E. S. (2010). Organizational Culture and Leadership.

[52] Nembhard IM, Edmondson AC (2006). Making it safe: the effects of leader inclusiveness and professional status on psychological safety and improvement efforts in health care teams. J Organ Behav.

[53] Aarons GA, Ehrhart MG, Farahnak LR, Sklar M (2014). Aligning leadership across systems and organizations to develop a strategic climate for evidence-based practice implementation. Annu Rev Public Health.

[54] Egeland KM, Skar AMS, Endsjø M, Laukvik EH, Bækkelund H, Babaii A (2019). Testing the leadership and organizational change for implementation (LOCI) intervention in Norwegian mental health clinics: a stepped-wedge cluster randomized design study protocol. Implementation Sci.

[55] Metz A, Kainz K, Boaz A (2023). Intervening for sustainable change: Tailoring strategies to align with values and principles of communities. Front Health Serv.

[56] Powell BJ, Beidas RS, Lewis CC, Aarons GA, McMillen JC, Proctor EK (2017). Methods to Improve the Selection and Tailoring of Implementation Strategies. J Behav Health Serv Res.

[57] Gifford WA, Davies BL, Graham ID, Tourangeau A, Woodend AK, Lefebre N (2013). Developing leadership capacity for guideline use: a pilot cluster randomized control trial. Worldviews Evid Based Nurs.

[58] Gifford WA, Davies B, Edwards N, Graham ID (2006). Leadership strategies to influence the use of clinical practice guidelines. Nurs Leadersh (Tor Ont).

[59] Ploeg J, Davies B, Edwards N, Gifford W, Miller PE (2007). Factors influencing best-practice guideline implementation: lessons learned from administrators, nursing staff, and project leaders. Worldviews Evid Based Nurs.

[60] Jorm AF, Korten AE, Jacomb PA, Christensen H, Rodgers B, Pollitt P (1997). "Mental health literacy": a survey of the public's ability to recognise mental disorders and their beliefs about the effectiveness of treatment. Med J Aust.

[61] World Health Organization. Health Literacy. Denmark; 2013. https://www.who.int/europe/publications/i/item/9789289000154.

[62] Crabb J, Stewart RC, Kokota D, Masson N, Chabunya S, Krishnadas R (2012). Attitudes towards mental illness in Malawi: a cross-sectional survey. BMC Public Health.

[63] Korhonen J, Axelin A, Stein DJ, Seedat S, Mwape L, Jansen R (2022). Mental health literacy among primary healthcare workers in South Africa and Zambia. Brain Behav.

[64] Kigozi FN, Ssebunnya J (2009). Integration of mental health into primary health care in Uganda: opportunities and challenges. Ment Health Fam Med.

[65] Kapungwe A, Cooper S, Mayeya J, Mwanza J, Mwape L, Sikwese A (2011). Attitudes of primary health care providers towards people with mental illness: evidence from two districts in Zambia. Afr J Psychiatry (Johannesbg).

[66] Feyissa GT, Balabanova D, Woldie M (2019). How Effective are Mentoring Programs for Improving Health Worker Competence and Institutional Performance in Africa? A Systematic Review of Quantitative Evidence. J Multidiscip Healthc.

[67] Anatole M, Magge H, Redditt V, Karamaga A, Niyonzima S, Drobac P (2013). Nurse mentorship to improve the quality of health care delivery in rural Rwanda. Nurs Outlook.

[68] Wainberg ML, Scorza P, Shultz JM, Helpman L, Mootz JJ, Johnson KA (2017). Challenges and Opportunities in Global Mental Health: a Research-to-Practice Perspective. Curr Psychiatry Rep.

[69] Galvin M, Byansi W (2020). A systematic review of task shifting for mental health in sub-Saharan Africa. Int J Ment Health.

