# Supplementary material for: Improving integrated depression and non-communicable disease care in Malawi through engaged leadership and supportive implementation climate
Source: BMC Health Serv Res. 2023 Dec 14;23:1413. doi: 10.1186/s12913-023-10344-7 (PMC10722817; doi:10.1186/s12913-023-10344-7)
Supplement: Supplementary file 1 — Additional file 1. Interview guide. [file 12913_2023_10344_MOESM1_ESM.docx]

**SHARP DMO Interview Guide** **v2.1 (1^st^ September, 2021)**

Hello, my name is [INTERVIEWER NAME], and I am working with researchers at UNC-Project in Lilongwe. We are conducting interviews with individuals who have been participating in the integration of depression services and NCD care. We are asking for your feedback so we can make improvements to the intervention for future use. No intervention or individual is perfect; please do not worry about hurting our feelings or the feelings of those you work with – I can ensure your information and responses will be kept confidential. If you found that something was not to your liking, please let us know so we can make the intervention better for NCD patients across Malawi.

As a reminder, you are not required to answer my questions, and you may skip any questions that make you uncomfortable. If you decide that you no longer want to participate in this interview, it will not affect anything regarding you status at this hospital or on our study. As a reminder, I will use a digital recorder to record our conversation.

Do you have any questions before we begin the interview?

**TURN ON DIGITAL RECORDER**

**Begin by stating: I am [INTERVIEWER NAME] interviewing participant [PARTICIPANT #] on [DATE] [START TIME].**

| **Topics and Main Questions** | **Probes** |
| --- | --- |
| 1. **Warm-up Questions** | |
| What is your current job role?  How long have you been working in this role?  Could you please tell me which services are provided to patients at this NCD clinic? | How do you prioritize these services and what makes you prioritize them that way? |
| 1. **Organizational Climate and Change** | |
| When the Ministry of health introduces new services such as depression care into routine clinical practice, additional work may be required by the providers without receiving additional compensation. How do you think most providers at this clinic feel about the additional work required by depression integration?  Some clinics have really been able to make depression integration work, whereby the providers at those clinics complete a PHQ-2 or a PHQ-9 with almost each and every patient.  Some NCD coordinators tasked with training and supervising their fellow providers on depression care have noted a challenge whereby provider turnover has resulted in those they trained no longer being available either because they have been transferred to a different clinic or facility or because they have left to attend a school program. What has provider turnover been like at this NCD clinic?  Sometimes, even NCD coordinators themselves can turnover – has that situation occurred at this clinic?  How informed would you say you are regarding the challenges to depression care integration within the NCD clinic?  As you know, NCD providers have been trained to screen for and treat depression. Some providers have told us that they really enjoy applying their new depression care skills even though it makes the clinic day last longer. What factors do you think make these providers to enjoy depression screening and treatment?  On the opposite side, despite receiving this training, some providers struggle to consistently screen and treat each and every patient. Does this NCD clinic face such difficulties?  Some of our earlier interviews found that a common screening challenge was due in-part to providers feeling overburdened with a high patient volume and just wanting to stop screening so they could get through the line, especially to help diabetes patients who have maybe come very early and without taking food.  What has been the impact of involving Friendship Bench Counselors in depression care at this clinic?  What plans are there to sustain depression integration program at the clinic? | How can provider attitudes be improved regarding depression care integration?  Whose job is it to change provider attitudes regarding depression care integration at this clinic?  What do you think makes this level of screening possible?  Is that level of screening possible at this clinic?  What would it take for providers at this clinic to complete a PHQ-2 or PHQ-9 with almost every patient?  What factors contribute the most to provider turnover?  How can the issue of provider turnover be addressed?  *Note: If DMO answers “yes” then ask:  If so, how was the situation handled?   1. What could have been done to improve the situation?   *Note: If DMO answers “no” then ask:  If not, what would the hospital do in the event that the current NCD coordinator announced they would be leaving in the coming two months to attend a school program?   1. How would that change affect the quality of depression integration?   Whose role is it to inform you of issues related to depression integration within NCD clinic?  What type of information would you need to determine how well depression integration is going at the NCD clinic?  What factors seem to be contributing the most to the difficulties regarding depression integration?  How do you think the patient volume affects providers’ attitudes regarding screening patients for depression at this NCD clinic?   1. Who has the power to improve their attitudes? 2. What would those improvements look like?   How will the SHARP study ending impact day to day integration of depression care at this clinic? |

| 1. **External Impacts on Implementation Strategy Delivery** | |
| --- | --- |
| How has the presence of the SHARP RA affect depression care integration at this clinic?  In what ways has the COVID-19 pandemic affected depression care integration at this clinic? | How has depression care integration been affected during period where the RA has worked away from the clinic?  Why do you think the RA has the effect that they do on this clinic?  How could depression care integration be improved right now, despite the challenges posed by the COVID-19 pandemic? |
| 1. **Conclusion** | |
| What else have we not yet discussed that you think is important for the research team to know about? |  |
| I want to thank you for your time today and for participating in our study. Your answers to our questions help us understand how to improve this program for the future. | |

**End by stating: I am [INTERVIEWER NAME] interviewing participant [PARTICIPANT #] on [DATE] [END TIME]**

**TURN OFF DIGITAL RECORDER**

**SHARP NCD Coordinator / Provider Interview Guide**

**v2.1 (1^st^ September, 2021)**

Hello, my name is [INTERVIEWER NAME], and I am working with researchers at UNC-Project in Lilongwe. We are conducting interviews with individuals who have been participating in the integration of depression services and NCD care. We are asking for your feedback so we can make improvements to the intervention for future use. No intervention or individual is perfect; please do not worry about hurting our feelings or the feelings of those you work with – I can ensure your information and responses will be kept confidential. If you found that something was not to your liking, please let us know so we can make the intervention better for NCD patients across Malawi.

As a reminder, you are not required to answer my questions, and you may skip any questions that make you uncomfortable. If you decide that you no longer want to participate in this interview, it will not affect anything regarding you status at this hospital or on our study. As a reminder, I will use a digital recorder to record our conversation.

Do you have any questions before we begin the interview?

**TURN ON DIGITAL RECORDER**

**Begin by stating: I am [INTERVIEWER NAME] interviewing participant [PARTICIPANT #] on [DATE] [START TIME].**

| **Topics and Main Questions** | **Probes** |
| --- | --- |
| 1. **Warm-up Questions** | |
| What is your current job role?  How long have you been working in this role?  Could you please tell me which services are provided to patients at this NCD clinic? | How do you prioritize these services and what makes you prioritize them that way?  How do you think most of your fellow NCD colleagues prioritize the services they provide at this clinic? Why do you think they prioritize them in that way? |
| 1. **Organizational Climate and Change** | |
| What are the issues at this clinic that may have contributed to how many patients are screened for depression on daily basis?  About how many patients do you think providers in this clinic treat on an average clinic day?  Please describe how your working relationship with the DMO has been during the time of depression care integration.  How aware do you think the DMO is of the challenges this clinic is facing regarding depression care integration?  How would you describe the coordinators attitude regarding depression care integration at this clinic?  If you could remove depression care from the services provided at this NCD clinic, would you do so?  During your time as a provider in the NCD clinic, about how many providers have left the clinic, either by been transferred to other departments within this hospital or to other facilities or gone to school?  During your time as a provider in the NCD clinic, about how many NCD coordinators have left the clinic, either by been transferred to other departments within this hospital or to other facilities or gone to school?  Have there been times in the past when providers at this clinic have been asked to integrate a new clinical practice into their day-to-day routines as providers?  What plans are there to sustain depression integration program at the clinic?  What has been the impact of involving Friendship Bench Counselors in depression care at this clinic? | How much does the coordinator influence providers at this clinic to screen each and every patient for depression?  Who (if anyone) has the power to influence providers at this clinic to screen each and every patient for depression?  What would need to change so that every provider could screen every patient they see at this clinic for depression?  How has this workload affected depression care integration?  Who has the power to impact the workload?  What could they do to improve the workload for providers?  How would you describe the DMO’s level of support regarding depression care integration at this clinic?  How does the DMO’s level of support affect depression care integration?  What would influence the DMO to be more supportive of depression care integration?  How does that level of awareness affect depression care integration?  How aware do you think the DMO is of the strengths this clinic shows regarding depression care integration?  How does that level of awareness affect depression care integration?  If you were the DMO at this hospital, what (if anything) would you do differently to make depression care integration more successful?   - What factors are keeping the DMO from taking the actions you just described?   How does the coordinator’s attitude affect depression care integration?  What is required to change the coordinator’s attitude regarding depression care integration?  If you were the coordinator at this hospital, what (if anything) would you do differently interact with the DMO to make depression integration more successful?   - What factors are keeping the coordinator from taking the actions you just described?   In your opinion should depression care integration be part of the NCD coordinators duties, why or why not?  Why or why not?  How have those providers leaving affected the integration of depression care?  What have you done to try and cope when providers leave the clinic?  What do you think is needed to improve the continuity of providers at this clinic?  How would better continuity of providers affect depression care integration?  *Note if at least one coordinator has left during the provider’s time in the NCD clinic, please ask:  How have coordinators leaving affected the integration of depression care?  What did the NCD clinic do to try and cope with that challenge?  What needs to happen to improve continuity of coordinators?  How successful was that integration?  What made that integration go on well?  What made it difficult? |

| 1. **External Impacts on Implementation Strategy Delivery** | |
| --- | --- |
| How has the presence of the SHARP RA affected depression care integration at this clinic?  In what ways has the COVID-19 pandemic affected depression care integration at this clinic?  Who at this clinic do you think is benefitting the most from depression care integration?  How have partner organizations such as UNC-Project in Lilongwe (or MEIRU in CH or KA) impacted depression care at this clinic?  How has the Ministry of Health affected depression care at this clinic? | What are the most important ways that depression care integration has been affected during period where the RA has worked away from the clinic?  Why do you think the RA has this effect on depression integration?  How would integration work if the RA were not there?  How could depression care integration be improved right now, despite the challenges posed by the COVID-19 pandemic?  Could you tell me more about that response?  *Note: Ask if responses indicate that other clinicians are benefit most…leave it if they say patients are the ones who are benefit most   - How does the perception that some individuals might be benefitting from depression integration impact depression integration at this clinic? |
| 1. **Conclusion** | |
| What else have we not yet discussed that you think is important for the research team to know about? |  |
| I want to thank you for your time today and for participating in our study. Your answers to our questions help us understand how to improve this program for the future. | |

**End by stating: I am [INTERVIEWER NAME] interviewing participant [PARTICIPANT #] on [DATE] [END TIME]**

**TURN OFF DIGITAL RECORDER**

**SHARP Research Assistant Interview Guide**

**v2.1 (1^st^ September, 2021)**

Hello, my name is [INTERVIEWER NAME], and I am working with researchers at UNC-Project in Lilongwe. We are conducting interviews with individuals who have been participating in the integration of depression services and NCD care. We are asking for your feedback so we can make improvements to the intervention for future use. No intervention or individual is perfect; please do not worry about hurting our feelings or the feelings of those you work with – I can ensure your information and responses will be kept confidential. If you found that something was not to your liking, please let us know so we can make the intervention better for NCD patients across Malawi.

As a reminder, you are not required to answer my questions, and you may skip any questions that make you uncomfortable. If you decide that you no longer want to participate in this interview, it will not affect anything regarding you status at this hospital or on our study. As a reminder, I will use a digital recorder to record our conversation.

Do you have any questions before we begin the interview?

**TURN ON DIGITAL RECORDER**

**Begin by stating: I am [INTERVIEWER NAME] interviewing participant [PARTICIPANT #] on [DATE] [START TIME].**

| **Topics and Main Questions** | **Probes** |
| --- | --- |
| 1. **Warm-up Questions** | |
| What is your current job role?  How long have you been working in this role?  Could you please tell me which services are provided to patients at this NCD clinic? | How do you think these services are prioritized at this clinic and what makes you prioritize them that way? |
| 1. **Organizational Climate and Change** | |
| How much of a challenge has it been for providers at this clinic to screen each and every patient?  What sort of an impact does the DMO have on depression integration at this clinic?  How aware do you think the DMO is of the challenges this clinic is facing regarding depression care integration?  How would you describe the coordinators attitude regarding depression care integration at this clinic?  If the providers at this clinic had the option to remove depression care from the services they provide, do you think most would do so? Why or why not?  The number of patients who attend on any given clinic day can often pose a challenge for the few providers who are scheduled on the rota. About how many patients do you think providers in this clinic treat on an average clinic day?  During your time working as an RA, about how many providers have left the clinic, either by being transferred to other departments within this hospital or to other facilities or gone to school?  During your time working on the SHARP study, about how many coordinators have left the clinic, either by being transferred to other departments within this hospital or to other facilities or gone to school? | How much power does the coordinator have to influence other providers at this clinic to screen for depression with each and every patient?  Who (if anyone) has the power to influence providers at this clinic to screen each and every patient for depression?  What would need to change so that every provider could screen every patient they see at this clinic for depression?  How does the DMO’s attitude affect depression care integration?  What is required to change the DMO’s attitude regarding depression care integration?  How much power does the DMO have to impact the behavior of providers at this clinic?  Who has the most power to impact provider behavior at this clinic?  How does that level of awareness affect depression care integration?  If you were the DMO at this hospital, what (if anything) would you do differently to make depression care integration more successful?   - What factors are keeping the DMO from taking the actions you just described?   How does the coordinator’s attitude affect depression care integration?  What is required to change the coordinator’s attitude regarding depression care integration?  How about if you were the Coordinator at this clinic, what (if anything) would you do differently to make depression care integration more successful?   - What factors are keeping the Coordinator from taking the actions you just described?   How has this workload affected depression care integration?  Who has the power to impact the ratio of patients to providers? What could they do to change the ratio?  How have those providers leaving affected the integration of depression care?  What has the clinic done to try and cope when providers leave the clinic?  What do you think is needed to improve the continuity of providers at this clinic?  How would better continuity of providers affect depression care integration?  How did it affect depression care integration when the coordinator role changed hands?  What are the biggest challenges when transitioning from one coordinator to another? What could improve these challenges?  What factors have made transitioning from one coordinator to another successful? Why do you think these efforts were successful? |

| 1. **External Impacts on Implementation Strategy Delivery** | |
| --- | --- |
| How do you think your presence in the clinic, as a SHARP RA, affected depression care integration? Why do you think you’ve had that affect?  In what ways has the COVID-19 pandemic affected depression care integration at this clinic?  Who at this clinic do most providers think is benefitting the from depression care integration?  Have there been times in the past when providers at this clinic have been asked to integrate a new clinical practice into their day-to-day routines as providers? If yes, can you please describe them?  What do you think will happen regarding depression care integration once the SHARP study ends? | How do you think depression care integration was affected during periods where you were called to work away from the clinic?  If you think back to the start, how was your role as the RA explained to the NCD providers at this hospital?  What do the providers at this clinic think your job entails?  How could your role have been different to decrease the impact you’ve had on depression integration?  How could depression care integration be improved right now, despite the challenges posed by the COVID-19 pandemic?  How have outside organizations like UNC-Project in Lilongwe (or MEIRU if interviewing in CH or KA) affected depression care at this clinic?   - To what extent to do providers at this clinic believe certain individuals might be benefitting more than others? Why do you think they think this? - How does the perception that someone individuals might be benefitting from depression integration affect integration efforts?   How has the Ministry of Health affected depression care at this clinic?  How successful was that integration?  What made that integration go on well?  What made it difficult?  How could the sustainability of depression integration be improved at this clinic? |
| 1. **Conclusion** | |
| What else have we not yet discussed that you think is important for the research team to know about? |  |
| I want to thank you for your time today and for participating in our study. Your answers to our questions help us understand how to improve this program for the future. | |

**End by stating: I am [INTERVIEWER NAME] interviewing participant [PARTICIPANT #] on [DATE] [END TIME]**

**TURN OFF DIGITAL RECORDER**
